# Time for change in implementation research and practice

**DOI:** 10.1186/s12916-026-04670-w

**Published:** 2026-02-02

**Authors:** Mike English, Jacob McKnight, Sassy Molyneux, Charles Vincent, Sebastian Fuller

**Affiliations:** 1 KEMRI-Wellcome Research Programme, Nairobi, Kenya; 2 Nuffield Department of Medicine, Health Systems Collaborative, University of Oxford, Oxford, UK; 3 Department of Experimental Psychology, University of Oxford, Oxford, UK

**Keywords:** Implementation, Intervention, Study design, Frameworks, Time, Temporal, Adoption

## Abstract

**Background:**

We argue implementation research pays insufficient attention to time. We were prompted by learning gained from the Harnessing Innovation in Global Health for Quality Care (HIGH-Q) programme to explore implementation through time as an analytical lens. Time directly underpins how individuals, teams, and organisations adopt and sustain new practices, yet existing frameworks primarily reference it indirectly. We propose that considering time as a multi-dimensional construct is relevant to the science of implementation in complex systems and to promoting its thoughtful practice.

**Arguments:**

HIGH-Q research involved coordinated ethnographic, quantitative and interventional studies of workforce enhancements in hospitals already benefiting from long-term neonatal technology and quality improvement support. Findings made it clear how time scarcity constrains improvement and use of new technologies in low-resource environments. New clinical technologies such as continuous positive airway pressure require time of users directly and indirectly linked to new cognitive and coordination work. Tasks compete for scarce time resulting in prioritisation, while time is needed for skill development, reflection, and team adaptation.

Conceptually we suggest the following: (1) time functions as a finite and negotiable resource that must be deliberately allocated to new practices, without creating temporal space, change efforts risk displacing existing essential work; (2) “hidden time” is required for reflection, collaboration, management and internalisation of new routines—activities rarely acknowledged in project planning; (3) time is an expression of value, reflecting what actors prioritise and the moral or organisational meaning attached to the allocation of effort; (4) healthcare work is governed by temporal structures—shifts, schedules, and social norms—that may hinder flexibility and adaptation; (5) maintaining “time in reserve” supports resilience and psychological recovery in stressful environments, yet interventions may erode this capacity; and (6) implementers’ own time investments are frequently omitted when characterising interventions, despite being crucial for sustainability.

**Conclusions:**

Viewing implementation through the prism of time exposes hidden constraints and misalignments between expectations, timelines and real-world conditions. Time in its multiple manifestations should be explicitly examined alongside theories of change and implementation frameworks to help understand why interventions in complex systems succeed or fail, especially where personnel and resources are already scarce.

## Background

Implementation research spans the scientific study of ‘the act of carrying an intention into effect’ including policies, programmes, or individual practices and how ‘to promote the systematic uptake of research findings and other evidence-based practices into routine practice’ [1]. It is needed to address the gap between an ever-increasing number of interventions and the provision of high-quality care to all [2]. As a field, implementation research is now associated with scientific methods increasingly emphasising the use of theory-informed intervention strategies and evaluation designs. However, we suggest that one potential contributor to the failure of interventions or challenges of later translation into routine settings has been under-appreciated. In this Debate, prompted by findings exploring a large improvement programme, we consider implementation through the explicit prism of time.

In Rogers’ foundational text on Diffusion of Innovations, the point is made that in much behavioural research ‘the time dimension is simply ignored’ [3]. Rogers explicitly considers time in reference to the information-decision process (for an individual or organisation), the relative earliness or lateness with which an innovation is adopted and the overall rate of adoption within the system under study [3]. We examined widely cited implementation science frameworks for their treatment of time and the TiDIER Checklist that aims to promote ‘the description of interventions in sufficient detail to allow their replication’ [4–10]. Normalisation Process Theory highlights that “the production and reproduction of a material practice requires continuous investment by agents in ensembles of action that carry forward in time and space”. The Non-Adoption Abandonment Scale-up Spread and Sustainability Framework reminds us of the need for “continuous embedding and adaptation over time” [10, 11]. Other frameworks remind us of the need for ‘dedicated time’ to promote, champion or give people an opportunity for adoption or implementation, the need to consider the time taken or number of times taken to implement, that time may be needed for local trials of the innovation and its adaptation and, of course, that implementation as a process may take time [5–10]. However, time is largely referred to when characterising other concepts rather than being a specific consideration in its own right.

Our interest in time emerged from multidisciplinary studies that contributed to the Harnessing Innovation in Global Health for Quality Care (HIGH-Q) research programme that involved several authors (and see acknowledgements). This explored how busy, stressful and under-resourced Kenyan hospitals respond to a major intervention. The NEST360 programme (Table 1) aims to upgrade hospital neonatal units so they can provide more specialised care to sick and preterm babies and, in our estimation, has largely successfully created a multi-stakeholder, multi-site network that could support effective change [12–14]. In further examining to what degree change was occurring at the frontline, ideas were generated that drew on our extensive HIGH-Q research (Panel 1) and were discussed and refined at a specially convened workshop (see acknowledgements). This allowed us to generate a set of time-related concepts that we feel may be useful to others involved in implementation research. Our aim is to complement, not replace, current frameworks and focus attention on time as a specific dimension when thinking about implementation within typically complex health systems.

**Table 1:** The Neonatal Essential Solutions and Technologies (NEST360) programme

Reducing neonatal mortality to a target level of 12/1000 livebirths became a specific Sustainable Development Goal in 2015. The NEST360 programme has been supporting 4 countries’ efforts to achieve this since 2019 by strengthening provision of non-tertiary (Level 2) newborn unit (NBU) care.(12) The NEST360 approach and rationale have been described in detail elsewhere but in brief include provision and long-term maintenance of multiple technologies, training of staff, coordination and mentorship of hospital QI teams and improving data systems that support both the QI and wider NEST360 audit and feedback systems.(13)	

*Panel 1: Insights from the Harnessing Innovation in Global Health for Quality Care (HIGH-Q) research programme that highlight time as an important dimension of implementation.*

HIGH-Q examined how extensive implementation efforts by the NEST360 programme might be affected by their resource limitations, with special attention paid to poor staffing. To assess workforce as a key mediator of NEST360 implementation aims for better neonatal care, we used quantitative observational methods, examined workforce enhancements and undertook extensive ethnographic work employing observation, interviews, shadowing and group discussions involving health care staff and families over 24 months [15]. Detailed findings are reported elsewhere. Here, we offer brief insights that prompted us to turn our attention to time.

- New care activities such as setting up and initiating continuous positive airway pressure (CPAP) or using other technologies consume time—but nurses were already unable to complete existing, expected care tasks in an environment with extremely limited nursing hours per patient per day [16].
- Time allocated for scheduled training to enable use of technologies provides foundational knowledge and skills, but building confidence and competence, both individually and within teams, to use technologies such as CPAP requires long-term continuous reflective practice, peer-to-peer support, co-ordination and collaboration, which are new and additional calls on time [17–19].
- When time is limited, ‘important’ tasks are done such as administering intravenous medicines and completing core aspects of paperwork, limiting the use of a new technology. Often, technical tasks seem prioritised over relational work such as time for effective communication as part of family-centred care, something strongly influenced by professional and practical norms [16–18, 20].
- Work schedules and routines limit flexibility and may preclude effective implementation of new tasks or practices (e.g. CPAP is very rarely initiated at night if a baby is born preterm). Implementation in a work setting is also navigated together with the demands and routines of non-working lives such as managing travel to work or attending important social events; these schedules and routines are rarely acknowledged by implementers but very much ‘in view’ of all those in the setting [21].
- When staff are relentlessly completing tasks while facing the everyday challenges of low resource environments, the capacity of individuals and teams for self-care, for example, after the death of a baby, is very limited. Interventions that increase work intensity may exacerbate this problem. Increasing time for self-care may then have a beneficial effect on implementation in the long term [18, 20].
- Activities driven by implementers based on external ideas of the time needed for change may not account for often dynamic local conditions; for example, the movement of personnel, resulting in dissonance between implementers’ timetables and recipients’ realities.
- Interestingly, in accounts of implementation, the working time of external teams who prepare for, conduct and provide follow-up support for supervisory or QI processes is rarely captured as a part of articulating the intervention to be sustained.

## Main text

### Time as an explicit dimension in implementation

Everyone is familiar with the ideas that ‘change takes time’ and in some cultures that ‘time is money’. It is therefore surprising that so little attention has been paid to time in a field of research that concerns itself with changing the behaviours of individuals, teams, organisations or institutions. However, even these two apparently simple sayings conceptualise time differently. We first consider time in its best recognised form, as a resource, and then explore aspects of time relevant to implementation that are perhaps paid less attention.

#### Time as a finite, measurable and exchangeable resource

Most interventions consume the time of providers, patients, carers or communities even if they ultimately aim to ‘save time’. *Time* *in this sense is seen as a finite and measurable resource.* In many cases, implementation efforts may either require that we create or free up time for those performing new or additional activities or behaviours. If we do not, we risk consuming the available stock of time. This may undermine the change we desire or reduce time for prior activities with potentially unintended negative consequences. *If time can be consumed or ‘spent’*, *it can also be saved*. Interventions may seek to streamline processes. Digital health implementation is often justified by claims of greater effectiveness and being ‘time-saving’ (not always realised [22]). Our typical expectation is that time saved will be used for another valued activity, improving quality and productivity. *Managerially*, *therefore, time available is the denominator allowing us to assess ‘productivity’* or the outputs produced in a fixed time in keeping with the tenets of Taylorism [23]. These ideas imply that as we design and conduct implementation studies, we should specifically anticipate impacts on health system staff time, both positive and negative, and their productive capacity. Although there are many methods for and a long history of assessing workers’ use of time, these rarely seem applied in current implementation research. Our experience and recent reports suggest that more attention should be paid to these issues [24, 25].

*If time is a resource, then who decides how it is allocated?* Making allocation decisions may require negotiation as much health care is provided by teams or more broadly by organisations. Many implementation interventions employ new tools or technologies. *These artefacts embody time.* Their use may directly consume time (even if they might save time overall) and they are often part of a sequence of events structured within time (see below). Decisions that affect whether time is allocated to an implementation activity will then depend on individual and collective expectations of whether there is sufficient time to employ the technique or tool. In fact, effective implementation may often require new forms of communication, coordination or collaboration for their success [11]. Implementers should therefore consider whose time is required and the conditions that constrain decision-making in favour of allocating time to desired behaviours. Seen like this, it becomes clear that *if time can be allocated or traded*, *it can also be invested****.*** Implementation may require an ‘up-front’ commitment of people’s working time or that individuals, teams or organisations contribute their own (non-work) time.

#### Hidden time for cognitive and relational work

Changing practice, using a new tool or technology, often requires explicit allocation of time as a resource, as outlined above, including for formal education or training. However, ***there is often considerable, unmeasured time that needs to be devoted to the process of normalising a new practice or technology***, as this requires individual and group reflection and learning [11]. How much of health systems staff and implementers’ time this consumes likely depends on the complexity of the change and the support given during the change process [10]. In many cases, learning, reflection and the process of normalisation (including trialability) will require more time in the early phase of implementation. This may span initial communication and stakeholder engagement activities expected to foster healthcare staff engagement and ownership that are often combined with feedback or additional support for implementation (e.g. supervision or mentorship) [13]. These require that actors have the cognitive ‘space and time’ for receiving, analysing and reflecting on information, sometimes collectively. Increasingly, implementers expect health system staff to review, interpret and reflect on regularly provided reports or to review dashboards [13]. All these forms of cognitive work at the individual, group and organisational level that promote change are rarely expressly identified as needs and may be a ‘hidden’ draw on time.

#### Time as an expression of value

If we think of time as a finite resource that actors have a discretionary ability to invest, then it is logical to consider the choices health systems staff make when engaging (or not) in implementation activities as a reflection of what they value. *Where implementation requires such actors to allocate time to a new activity*, *then trade-off decisions are made* reflecting priorities and the perceived balance of benefits and harms of allocating time to different courses of action. These decisions may be influenced by individuals’ wider (and non-working) roles and responsibilities, their personal values, the pressure exerted on them to change and the values and culture of the organisations and institutions within which they work [26, 27]. *In some cases, the time spent on implementation may even be valued in moral terms.* Implementers often assume adopting their proposed change is for the good, as likely to generate organisational or health benefits. The time and effort invested by different stakeholders in any change may depend on what they consider to be a benefit, perceived opportunity costs, the immediacy with which benefits are seen and how much future benefits are discounted. Thus, one’s values or moral stance may countenance against actions required by implementers, resulting in failure to allocate or withdrawal of time and effort.

#### Time in reserve, enabling resilience

Implementers mostly consider the success of an intervention as the transition to a steady state in which new activities become embedded within a system running at full capacity and so apparently maximising productivity. We rarely imagine these systems as needing any reserve capacity. In most settings, however, systems benefit if individuals, teams and organisations have the ability to rapidly mobilise reserves including time to avoid poor performance or harms and enable health systems staff to deal proactively with acute or long-term stresses within their context [28]. Estabrooks defines these reserves as organisational slack; the ‘cushion of actual or potential resources which allows an organisation (unit) to adapt successfully to internal pressures for adjustments or to external pressures for changes’ [29]. Therefore, implementors need to carefully consider how the changes they desire affect work intensity and the time in reserve needed to manage unpredictability, acute and chronic stresses, and enable resilience. *The slack in the system required to accommodate new or changed work must often be in the form of staff time* [29, 30].

Moreover, in health care settings, under pressure, difficult working conditions and patients’ distress commonly cause health worker stress [31]. To limit the moral distress and burnout that can result, *health workers may require individual or collective ‘time-out’*, often also considered non-productive. Implementation efforts that increase work intensity may limit the time available for the continuous psychological running repairs that individuals and teams need. This can undermine whole system performance and, thus, any anticipated benefits of intervention.

#### Time and temporal structuring

*Human activities are often governed or structured by time* [32, 33]. Consequences of it being day or night, a weekday or weekend are familiar to us all. Surprisingly, however, implementation efforts and the research accompanying it may pay this aspect of time little attention, although healthcare work is subject to well-established schedules or routines that may powerfully influence organisational, individual or potential recipient behaviours. Additional temporal structuring of work may be driven by entrenched workplace or practical norms [26, 34]. Those planning or implementing interventions also pay limited attention to *how ‘non-work’ time may influence those expected to enact a change*. Yet what happens in and outside work in the lives of health system staff can impact our interventions in multiple ways, perhaps most directly through (un)willingness to adjust routines, schedules or the level of effort required to accommodate new practices. Even more broadly, how rigidly temporal structuring manifests itself may be influenced by varying socio-cultural views of time (and timeliness) itself [26].

#### Time for the work of implementers

Interventions and implementation strategies are often designed with variable levels of engagement from (and require the time of) system or organisational stakeholders, patient or community groups. If the desired changes in activity or behaviour are considered to be simple, implementers may only invest their time and stakeholder time in low-intensity inputs over relatively short periods. Where interventions are considered complex, implementation inputs may need to be of higher intensity and over longer periods [35]. In both cases, implementation activities are often clustered near the onset of a project before diminishing to a fixed, implementer-defined endpoint. *The planned time course often suits the implementers rather than respecting the time of stakeholders and those targeted by change efforts.* Furthermore, in many accounts of interventions, even in process evaluations, *the time input of implementers is often ignored* so that implementation effort and resources are not fully characterised [4, 36]. This is despite increasing recognition that the time and effort of implementing personnel are often important factors in the success of studied interventions and that sustaining or spreading successful change after any research may depend on similar additional contributions of time and effort [37].

Implementers are also typically subject to the temporal structuring outlined above, with their time governed by project plans, milestones, budgets, reporting schedules and more. Such temporal constraints may influence the work they do, their level of ambition, their urgency to achieve their aims and therefore their plans for change [38]. Both implementers and health system staff will also have to consider how their plans for change might accommodate, adapt to or disrupt the routines, schedules and rhythms of the settings subject to intervention and the impact of these choices [38].

#### Time as the causal progression of events

Often implicit in intervention thinking is that the effort of the implementers transforms forever the routine work of health system staff. This may require healthcare providers to learn and reliably exhibit skills that take time and practice to develop, including learning how to make careful decisions that reflect precise circumstances ‘in the moment’. The time course of these individual, team and organisational changes may vary, especially if complementary changes in skills or practice are required by multiple team members and at different levels of the system, while changes in complex systems will also depend on the system history reflecting ‘path dependency’. Implementers are becoming more used to laying out the changes they anticipate are needed in logic models, theories of change or programme theories. But these rarely articulate assumptions for how much time may be required for changes in individual or collective behaviours in the active implementation phase or the time required for changes to achieve a permanent system reset. Moreover, these representations of the expected causal sequence of events, while important, cannot fully account for system complexity [35]. *Implementers may therefore need to consider more explicitly time and complexity in theories of change and requirements for sustainable change.*

## Conclusions

The ideas outlined remind us that implementation success will depend on health system staff’s ability and willingness to invest and flexibly deploy their time as a resource to meet (our/implementer) project goals. Their working and non-working time is governed by visible schedules and routines and less visible norms that may influence changes many implementers hope to achieve. Collectively, these factors and the direct inputs of implementers’ time and effort will affect the time course and success of change efforts. More broadly, the settings targeted for implementation, even if apparently well-circumscribed, will typically manifest the properties of complex adaptive systems; being open systems subject to multiple wider influences, being made of multiple interacting elements with patterns of behaviour driven by their history, and having properties that include self-organisation and emergence [39]. Those intervening in such settings should recognise that health system staff and implementers themselves need time for reflection, learning, socialising changes and for coping with fluctuating work intensity while maintaining well-being. Both parties are thus engaged in managing complexity, including being alert to and mitigating unintended, possibly harmful consequences. Successful implementation then depends on how we take strategic account of time and the collective abilities of implementers and health system staff to link synergistically the administrative, adaptive and enabling approaches to leadership that may be needed when working within complex systems [40].

**Fig. 1:**
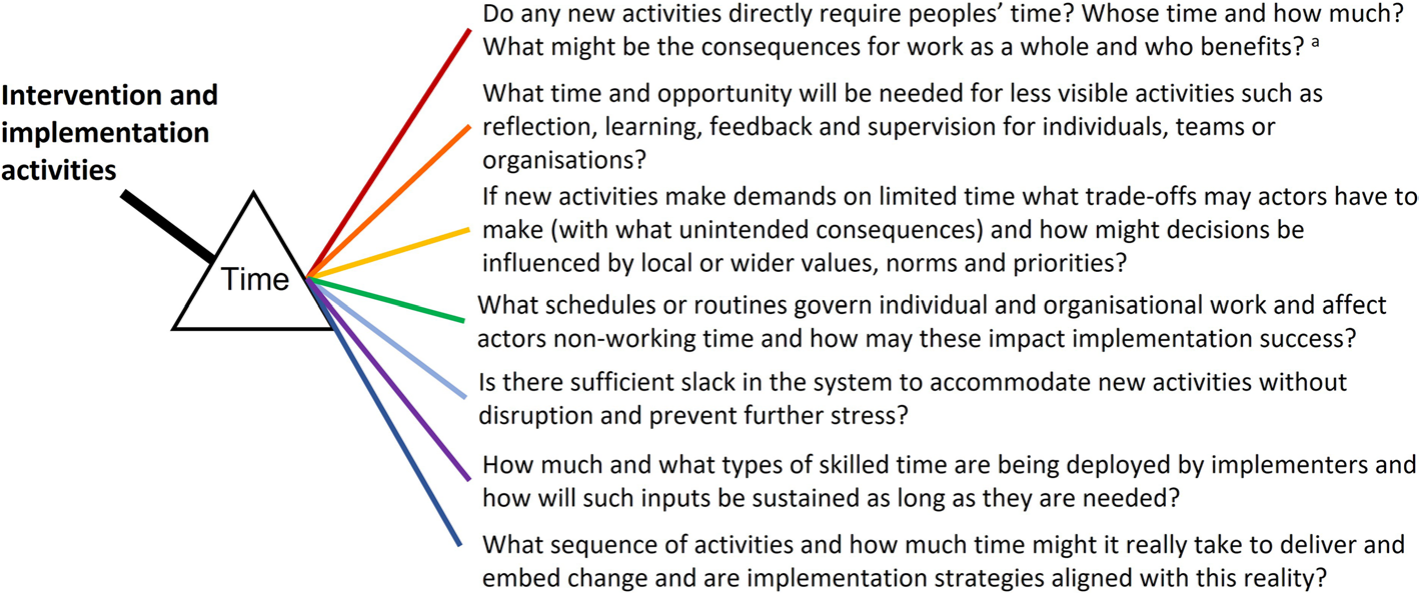
Viewing intervention through the prism of time—key questions for implementers. To address these questions, in addition to a review of relevant literature, formative work that helps uncover how work is done, existing working patterns and pressures, local norms and priorities and that helps anticipate possible challenges will likely be useful [41]. The aim is to uncover implicit knowledge of the system of interest. Work might include mapping existing and any new activities/processes, who they might impact and how, using observations and interviews and possibly co-design activities in addition to more formal time and motion assessments where appropriate [42]. More specific exploration of organisational slack might focus on whether there are sufficient staff to deliver quality of care prior to and after any intervention, whether staff have enough time for patients’ clinical and emotional needs and whether they have sufficient space and material resources to support any new implementation requirements [29]. Carefully articulated Theories of Change/Programme Theories that include the work, skill-mix and time of implementers and what elements of this will be required to sustain interventions should be produced. These should also articulate and justify the timing of implementation elements, how these interface with the rhythms of activity within the intervention settings and whether it is reasonable to expect the degree of change expected in the time allocated to the intervention [38]

Our HIGH-Q work strongly suggests that implementation failure or unintended consequences are likely in settings where the changes required are substantial and the stock of health system staff time is already severely constrained. Shining a light on time therefore seems a helpful adjunct to the use of recent theories and frameworks. We recommend implementation researchers and practitioners think about time’s multiple facets as they plan, deliver and evaluate interventions (Fig. 1). To this end, it may be useful to ask additional questions as we consider our plans and how they might interact with the complex contexts we often target for change, and as we characterise our roles as implementers so we are better equipped to identify what may be needed to sustain and scale up successful implementation strategies.

## Data Availability

No datasets were generated or analysed during the current study.

## Abbreviations

HIGH-Q: Harnessing Innovation in Global Health for Quality Care
CPAP: Continuous positive airway pressure
NBU: Newborn Unit
